# Preoperative localization of hyperfunctioning parathyroid glands with 4D-CT

**DOI:** 10.1007/s00405-015-3509-9

**Published:** 2015-03-14

**Authors:** Anke Katrin Lundstroem, Waldemar Trolle, Christian Hjort Soerensen, Peter Sand Myschetzky

**Affiliations:** 1 Department of Radiology, Copenhagen University Hospital Herlev, Herlev Ringvej 75, 2730 Herlev, Denmark; 2 Department of Head and Neck Surgery, Copenhagen University Hospital Rigshospitalet, Blegdamsvej 9, 2100 Copenhagen, Denmark; 3 Department of Radiology, Copenhagen University Hospital Gentofte, Niels Andersens Vej 65, 2900 Hellerup, Denmark

**Keywords:** Four-dimensional computed tomography, Primary hyperparathyroidism, Parathyroidectomy, Imaging

## Abstract

Primary hyperparathyroidism (pHPT) is almost exclusively the result of a solitary parathyroid adenoma. In most cases, the affected gland can be surgically removed, but precise preoperative imaging is essential for adenoma localization prior to surgical intervention. In this study, we evaluated the diagnostic value of four-dimensional computed tomography (4D-CT) as a preoperative imaging tool in relation to the localization of pathologic parathyroid glands in patients with pHPT and negative sestamibi scans. This study included 43 consecutive patients with pHPT referred for parathyroidectomy at the Department of Head and Neck Surgery of Copenhagen University Hospital Rigshospitalet in 2011 and 2012. All patients had a 4D-CT performed prior to parathyroidectomy. CT localization of the suspected adenoma was correlated to the actual surgical findings and subsequent histological diagnosis was also available as references for the accuracy of this imaging tool. Hyperfunctioning parathyroid glands were found in 40 patients. 4D-CT identified 32 solitary hyperfunctioning parathyroid glands located on the correct side of the neck (PPV 76 %) and 21 located within the correct quadrant (PPV 49 %). Unilateral resection was performed in 72 % of patients due to the localization findings of preoperative imaging. 4D-CT can, therefore, be considered an effective method for the preoperative localization of parathyroid adenomas and is an important tool in surgical intervention for patients referred to parathyroidectomy.

## Introduction

Primary hyperparathyroidism (pHPT) is defined as an autonomous overproduction of parathyroid hormone (PTH). This overproduction is in approximately 80–90 % of cases caused by a solitary parathyroid adenoma and in most other cases by diffuse hyperplasia, rare multiple adenomas or carcinoma [1]. Parathyroidectomy is the only curative treatment for pHPT, whether caused by adenoma or hyperplasia. The conventional surgical method consists of bilateral neck exploration with identification of all four parathyroid glands and resection of hyperfunctioning glands with pathological appearance [2]. In a majority of patients with pHPT, unilateral and focused surgical approaches are sufficient. Benefits of minimizing the extent of the surgical intervention are reduction in surgical trauma giving fewer complications, better cosmetic results, shorter hospitalization and convalescence, and better postoperative conditions for potential future operations in the thyroid region [1, 3–5]. To guide these focused surgical interventions, precise preoperative imaging is essential, showing the accurate localization of abnormal parathyroid glands. The commonly used parathyroid imaging technique is technetium-99 m sestamibi scintigraphy often complemented by ultrasound and in some cases CT or MRI [6]. Sestamibi scans are false negative or inconclusive in approximately 20 % of all patients, resulting in the need for other diagnostic imaging methods [3, 7, 8]. In 2006, a relatively new preoperative imaging modality was introduced called four-dimensional computerized tomography (4D-CT) [9]. 4D-CT is made up of a series of CT scans taken at different stages of enhancement and washout of contrast media in the parathyroid glands. In addition to a three-dimensional presentation of the anatomical localization of the glands, 4D-CT includes a time-dependent contrast enhancement of the gland as the “fourth dimension”. The fact, that the parathyroid glands and parathyroid adenomas receives contrast-enhanced blood a few seconds earlier than the thyroid tissue combined with a slightly higher flow, makes the adenomas to “lighten up” earlier the normal thyroid tissue, and also washes the contrast out earlier. That is the reason for the time-related re-scan of the area. If you look at the arterial anatomy, all four normal parathyroid glands get their arterial from the first side-branch of the thyroid arteries, this may explain why they receive the contrast a few seconds before the rest of the thyroid gland. The aim of this study was to evaluate the diagnostic value of 4D-CT as a preoperative imaging tool in relation to the localization of pathologic parathyroid glands in patients with pHPT and negative sestamibi scans.

## Patients and methods

This retrospective study includes all patients that in 2011 and 2012 were referred to the Department of Head and Neck Surgery at the Copenhagen University Hospital Rigshospitalet for surgical treatment of biochemically proven primary hyperparathyroidism, as confirmed by ionized plasma calcium levels >1.32 mmol/L and not suppressed PTH. All patients presented with negative sestamibi scans and subsequently underwent preoperative 4D-CT scans at the Department of Radiology at Copenhagen University Hospital Gentofte, followed by elective parathyroidectomy in the period from March 2011 to November 2012.

### 4D-CT scan technique

4D-CT was performed with 64-slice multidetector scanner (Philips Brilliance) with each examination consisting of five continuous helical acquisitions from the mandibular angle to 2 cm below the most inferior pulmonary apex. After precontrast scan, 100 ml non-ionic contrast medium was injected (Omnipaque 300 mgI/mL, GE Healthcare) with 3.5 mL/s via intravenous access in the right cubital vein, followed by arterial, portal vein, venous and late venous contrast helical series, respectively, 22, 52 s for 82 and 122 s after the beginning of contrast injection. Scan parameters: 120 kV, 200 mAs, pitch 0.671, rotation time 0.75 s, slice thickness 1 mm, increment 0.5 mm collimation 64 × 0.625, window (C/W) 40/350. All 4D-CT scan images were evaluated on a diagnostic screen and described by an experienced specialist in diagnostic radiology. The pathological parathyroid glands were identified by their pattern of time-related contrast enhancement and anatomical structure, mainly the size. Scan results were preoperatively discussed with the surgeon.

### Surgical procedure

All surgical procedures were carried out by one of the two specialists experienced in parathyroid surgery. Abnormal parathyroid glands were removed based on the surgeon’s assessment of gland size and shape. After removal of a gland with pathological appearance, an intraoperative parathyroid hormone (IOPTH) measurement was used to confirm the complete resection of abnormally secreting parathyroid tissue. IOPTH decrease >50 % within 10 min after resection suggested confirmation in the removal of the hyperfunctioning parathyroid tissue [10]. All dissections were initiated unilaterally corresponding to the quadrant where the 4D-CT revealed a suspicious process. In cases where an abnormal parathyroid gland was seen and removed, an IOPTH measurement was performed. If IOPTH decreased sufficiently, the surgery was finished. If either the 4D-CT described gland was found with normal appearance, or an insufficient fall in the IOPTH measurement was reported after excision, the surgical exploration was expanded until either a gland with pathological appearance was found and removed resulting in a subsequent sufficient IOPTH drop, or until all four quadrants were explored.

### Histopathological analysis and biochemistry

Preoperative and postoperative serum calcium and PTH were tested. Surgically removed parathyroid glands were weighted and histopathologically examined to confirm hyperfunctioning parathyroid tissue.

### Analysis

Diagnostic accuracy measures were used to evaluate the clinical performance of 4D-CT imaging studies by comparing the results of 4D-CT with peroperative findings and the histopathological diagnosis as a reference standard. Localization of suspicious parathyroid glands on 4D-CT and of surgically removed glands was reported as side (right or left) and quadrant (right upper, right lower, left upper, left lower) of the neck. The standard of reference for the final anatomic location of histologically hyperfunctioning parathyroid gland was the site and quadrant determined at surgery.

4D-CT imaging results were classified as true positive, false positive or negative based on correlation with anatomical findings at surgery and the histopathological results.

## Results

### Patient characteristics

Out of a total of 263 patients with pHPT, which were referred to the Department of Head and Neck Surgery at the Copenhagen University Hospital Rigshospitalet in 2011 and 2012, 43 consecutive patients (16 %) with negative or inconclusive sestamibi scan results were included in this study (31 women and 12 men, aged 44– years, mean 65). Three patients had previously undergone neck surgery, one parathyroidectomy, one hemithyroidectomy and one total thyroidectomy followed by postsurgical radioactive iodine treatment. 16 patients had undergone ultrasound before being referred to the Department of Head and Neck Surgery, reporting changes suspicious for parathyroid adenoma in six cases. In four patients, the ultrasound located the abnormal gland on the correct side of the neck, of which only one correct quadrant was reported.

### 4D-CT scan

**Fig. 1:**
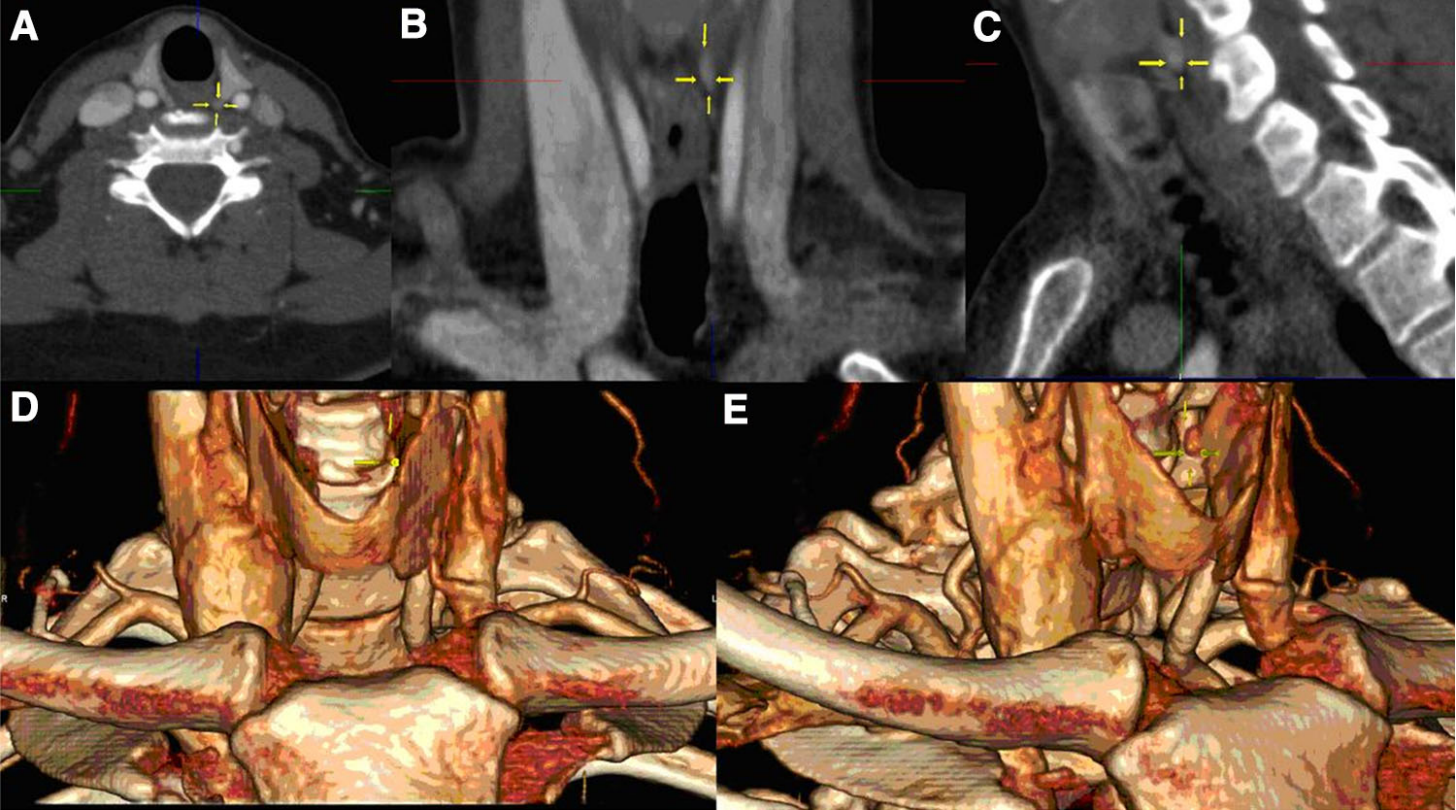
Example of preoperative localization of pathological parathyroid tissue with 4D-CT scan. Demonstration of an area suspicious for a parathyroid adenoma (*yellow arrows*) in the *left upper* quadrant in **a** axial, **b** coronal and **c** sagittal plane, and **d**, **e** three-dimensional reconstruction

4D-CT identified nodules suspicious for parathyroid adenoma in all 43 patients. In 31 cases, one nodule suspicious for a solitary adenoma was found on 4D-CT (for example, see Fig. 1). In seven cases, 4D-CT described two suspect findings, with one much more suspicious than the other, so that only the most suspicious was considered true. Only the most suspect finding was considered as a true positive. In four patients, the 4D-CT showed multiple (>2) suspicious nodules on one side of the neck. Only one 4D-CT scan was inconclusive with several questionable suspicious areas bilaterally (see Table 1).

**Table 1:** Findings of 4D-CT, surgery and histopathology

Number of suspect nodules on 4D-CT	Inconclusive	1	2	>2
Number of patients	1	31	7	4
Number of glands excised per patient	0	1	2	3
Number of patients	1	29	11	2
Number histopathologically verified HFPGs excised per patient	0	1	2	>2
Number of patients	3	38	2	0

*HFPG* hyperfunctioning parathyroid gland

### Surgery

The surgeons excised parathyroid glands with pathological appearance in 42 of 43 patients. In one patient previously treated with total thyroidectomy and radioactive iodine, no abnormal parathyroid gland could be detected. One suspicious gland was removed in 67 % of patients, 2 glands in 26 % of patients and 3 glands in 5 % of the patients (see Table 1). IOPTH measurements were performed during all surgical procedures, except one due to equipment failure. In 95 % of the patients, there was found a sufficient IOPTH drop after removal of all suspicious glands indicating adequate resection of hyperfunctioning parathyroid glands. There were no postsurgical complications such as hemorrhaging, wound infections or N. recurrens paresis, as investigated by post surgical fiberoptic laryngoscopy. In 31/43 (72 %) of the patients, the extent of the surgical intervention could be reduced to unilateral exploration.

### Histopathological analysis and biochemistry

The histopathological examination of the surgically resected parathyroid glands, confirmed the removal of a single hyperfunctioning gland (HFPG) in 88 % (38/43) of the patients. Two patients had multiglandular disease with two hyperfunctioning glands. In two patients were all removed parathyroid glands found to be histologically normal (see Table 1).

67 % of the surgically removed and histologically verified HFPGs were weighed after surgery with a median weight of 542 mg (range 85–2048 mg). 61 % of these HFPGs weighed less than 600 mg and 39 % more than 600 mg. 76 % of the HFPG weighing less than 600 mg and 91 % of the HFPG weighing more than 600 mg were on preoperative 4D-CT found on the correct side of the neck (see Table 2). All patients had presurgical mild hypercalcemia with slightly elevated plasma ionized serum calcium levels (mean 1.47 mmol/L, range from 1.34 to 1.61 mmol/L; normal 1.18–1.32 mmol/L) and no parathyroid hormone suppression (presurgical PTH plasma concentration: mean 23.39 pmol/L, range from 5.55 to 76.00 pmol/L; normal 1.18–8.43 pmol/L). Postsurgical were 91 % patients normocalcaemic, with a follow-up time of at least 1 year. The patient that underwent reoperation and one patient that got removed one adenoma and two histologically normal glands had relative hypoparathyroidism with mild hypocalcaemia and PTH in the normal range after the last surgery. The patient without finding of abnormal glands and one of the patients that only got removed histologically normal glands showed postoperative pHPT with hypercalcaemia and not suppressed PTH. The other patient that only got removed a histologically normal parathyroid gland was normocalcaemic after surgery, maybe because of compromised blood supply to an adenoma.

**Table 2:** Weight of hyperfunctioning parathyroid glands (HFPG) in patients with primary hyperparathyroidism according to sidelocalization on preoperative 4D-CT

		Sidelocalization on 4D-CT	
		Correct	Wrong
Weight of HFPG (mg)	542 ± 441	586 ± 464	204 ± 49
HFPG <600 mg	61 %	76 %	24 %
HFPG >600 mg	39 %	91 %	9 %

Data of the weight of HFPG shown are mean ± SD. Data for individual parameters were not available in all cases; data for parathyroid weight include 26 parathyroid adenomas

### Overall performance

Among the 43 patients undergoing 4D-CT imaging and parathyroid surgery, where hyperfunctioning parathyroid glands were identified in 40 patients (solitary pathologic glands in 38 patients and double hyperfunctioning glands in 2 patients). 4D-CT imaging was clearly positive for nodules in 42 patients. 32 solitary hyperfunctioning parathyroid glands, had on preoperative 4D-CT, been detected on the correct side of the neck (positive predictive value of 76 %) and 21 of these in the correct quadrant (positive predictive value 49 %) (see Table 3). In both patients with double hyperfunctioning parathyroid glands, one of the two pathological glands was identified in the correct quadrant on 4D-CT.

**Table 3:** Results of preoperative localization diagnostic with 4D-CT scan of hyperfunctioning parathyroid glands according to surgical findings in 43 patients with primary hyperparathyroidism

Surgical findings	# Pt.	4D-CT findings			
		True positive	False positive		Negative
		Correct localization (side/*quadrant*)	Wrong localization (side/*quadrant*)	Solitary HPFG in patient with double or no HFPG	No HPFG or inconclusive
Solitary HFPG	38	32 (84 %)/*21 (55* *%)*	5 (13 %)*/16 (42* *%)*	NA	1 (3 %)
Double HFPG	2	0	NA	2 (100 %)	0
No HFPG	3	NA	NA	3 (100 %)	0
Total	43	32 (74 %)/*21 (49* *%)*	5 (12 %)/*16 (37* *%)*	5 (12 %)	1 (2 %)
Likelihood of positive 4D-CT (4D-CTs with finding of HFPG/4D-CTs performed)					42/43 (98 %)
Likelihood of correct positive side/*quadrant* localization on 4D-CT					32/43 (74 %)
(4D-CTs with correct side/*quadrant* localization of HFPG/4D-CTs performed)					*21/43 (49* *%)*
Positive predictive value of correct side/*quadrant* localization with 4D-CT					32/42 (76 %)
(4D-CTs with correct side/*quadrant* localization of HFPG/4D-CTs with finding of HFPG)					*21/42 (50* *%)*

Italics values are the results regarding quadrant localization, while the numbers not in italics are those regarding side localization

*HFPG* hyperfunctioning parathyroid gland, *#Pt.* number of patients, *NA* not Applicable

## Discussion

In our study, four-dimensional computed tomography (4D-CT) was a potent instrument to localize most of the solitary hyperfunctioning parathyroid glands (HFPGs) in patients with primary hyperparathyroidism (pHPT) and negative sestamibi scintigraphy, so that the majority (72 %) of patients included could be operated unilateral. Standard surgical treatment of pHPT may routinely consist of bilateral neck exploration, including exposing all four parathyroid glands and excision of pathological appearing glands [5]. This invasive procedure may now be reduced to focused unilateral neck surgical exploration [5]. This is possible due to the performance of preoperative localization imaging of hyperfunctioning and enlarged parathyroid glands in combination with IOPTH measurement [5]. Standard imaging modalities used in Denmark are currently sestamibi scintigraphy, possibly in combination with SPECT (single photon emission computed tomography), ultrasound (US) and more infrequently computed tomography (CT). According to literature, approximately 20 % of sestamibi scans are false negative or inconclusive, despite biochemical evidence of primary hyperparathyroidism [3, 7, 8, 11]. In our study, sestamibi scan was negative in 16 % of the patients with pHPT referred to our surgical department. 4D-CT can be an ideal imaging alternative in these types of cases.

Also other recent studies have shown that 4D-CT is of value in the presurgical localization of hyperfunctioning parathyroid glands (HFPGs) [6, 9, 12–18], with an accuracy of localization ranging from approximately 73–93 % [19]. In our study, 84 % of the solitary pathological glands could be localized with preoperative 4D-CT on the correct side of the neck (PPV 76 %) and 55 % in the correct quadrant (PPV 50 %). One possible reason for the lower success in quadrant localization compared to side localization may result from different head positioning between CT examination and surgery. Another explanation could be that 4D-CT examination, as ultrasound, is heavily dependent upon the radiologist’s expertise. Depending on the structure, size and position of the thyroid gland, determination of the precise location between the upper and lower quadrant can be difficult.

4D-CT appears to be better than sestamibi scan and US for small adenomas and in cases of mild hypercalcemia [17, 20]. It has been described that the well-known inability of sestamibi to consistently identify small HFPGs may be due to the fact that the level of sestamibi uptake corresponds to the size and weight of the parathyroid glands, with a proposed cutoff of 600 mg proposed [20]. In our study, all patients had presurgical mild hypercalcemia with slightly elevated plasma ionized serum calcium levels (mean 1.47 mmol/L, range 1.34–1.61 mmol/L). Median weight of the HFPGs that were excised in our study was 542 mg with a range of 85–2,048 mg and 61 % weighing less than 600 mg. In our study, 76 % of the HFPG weighing less than 600 mg and 91 % of the HFPG weighing more than 600 mg were found on 4D-CT on the correct side of the neck. Thus, however side localization was a little better in case of heavy adenomas, 4D-CT could identify the majority of the small HFPG not found on sestamibi scintigraphy. Despite this fact, we observed that the evaluation of characteristic contrast enhancement in very small adenomas can be difficult, possibly leading to misinterpretation of lymph nodes as small adenomas on 4D-CT. But as this technique is new, the radiologist had no possibility for obtaining prior experience in evaluation of the 4D-CT. We can expect further improvement in the sensitivity and specificity of this new modality comparable with the increasing experience in interpreting 4D-CT scans; however, an inter-reader comparison is suggested with future evaluation of this technique.

The complication rates from surgery for pHPT increase significantly in reoperation. Thus, 8 % N recurrens paresis and 13 % permanent hypoparathyroidism are expected in cases of reoperation [21–23]. Therefore, good preoperative imaging and minimization of the extent of the surgical intervention is important. In our study, there was no N. recurrens paresis. Only one patient underwent reoperation and had relative hypoparathyroidism with mild hypocalcaemia and PTH within the normal range after the second surgery. The overall success rate of >90 % in surgical intervention corresponds to the best international results [17, 24]. A potential surgical operator limitation of this study is that the surgeons’ own visual assessment of the parathyroid glands appearance determines if the gland is removed or not. In some cases, the absences of a visual pathohistological appearance lead to nonexcision of the tissue which had been indicated by the 4D-CT study suggesting a pathological state smaller than can be seen by the operator.

Regarding cost and time effort of 4D-CT compared to sestamibi, the costs are approximately the same and 4D-CT takes less time [25]. In a survey of economic conditions of routine preoperative 4D-CT is concluded that the use of 4D-CT is considered to be a little more expensive than surgery without using preoperative 4D-CT, but that more patients can settle for minimally invasive procedures and shorter hospitalization [26].

The estimated radiation dose in 4D-CT and sestamibi scintigraphy are, depending on protocols in different institutions, respectively, 5.56–10.4 and 3.33–7.8 mSv [25, 27]. Although a 4D-CT study is considered to yield a higher dose of radiation as compared to a sestamibi scan, the average background radiation exposure of 3 mSv/y, and added exposures of less than 15 mSv are considered low risk for carcinogenesis [25]. However, the estimated dose to the thyroid gland is much higher performing a 4D-CT scan than a sestamibi scintigraphy [27]. Therefore, it is currently recommended to perform 4D-CT only in exceptional cases in patients under 40 years of age. The youngest patient in our study was 44 years. Newer CT scanners introduced at Copenhagen University Hospital Gentofte within the past year reduce radiation doses by up to 80 % and establishment of these systems nationally will ensure that the radiation dosages for all patients can be expected to fall drastically within the near future.

Most previous studies evaluating 4D-CT imaging are retrospective case studies and include a selected group of patients. For the use 4D-CT as the primary preoperative imaging modality, further prospective studies are required. One prospective study comparing preoperative sestamibi scan with 4D-CT is currently ongoing in Copenhagen at the Department of Radiology and Nuclear Medicine at University Hospital Gentofte in collaboration with Department of Endocrinology at University Hospital Herlev and the Department of Head and Neck Surgery at University Hospital Rigshospitalet.

## Conclusion

In patients with primary hyperparathyroidism and negative sestamibi scintigraphy, 4D-CT imaging before parathyroidectomy in combination with intraoperative parathyroid hormone measurement can be considered as useful diagnostic tool to localize hyperfunctioning parathyroid glands, supporting focused surgical intervention as presented in the study. However, the use of 4D-CT as the primary imaging modality will require further studies.
